# Pharmacokinetics and safety of ixazomib plus lenalidomide–dexamethasone in Asian patients with relapsed/refractory myeloma: a phase 1 study

**DOI:** 10.1186/s13045-015-0198-1

**Published:** 2015-09-04

**Authors:** Neeraj Gupta, Yeow Tee Goh, Chang-Ki Min, Jae Hoon Lee, Kihyun Kim, Raymond S. M. Wong, Chor Sang Chim, Michael J. Hanley, Huyuan Yang, Karthik Venkatakrishnan, Ai-Min Hui, Dixie-Lee Esseltine, Wee Joo Chng

**Affiliations:** Clinical Pharmacology, Millennium Pharmaceuticals, Inc., a wholly owned subsidiary of Takeda Pharmaceutical Company Limited, Cambridge, MA USA; Singapore General Hospital, Singapore, Singapore; Seoul St Mary’s Hospital, Seoul, South Korea; Gachon University Gil Medical Center, Incheon, South Korea; Samsung Medical Center, Sungkyunkwan University School of Medicine, Seoul, South Korea; Department of Medicine and Therapeutics, Prince of Wales Hospital, Sir Y. K. Pao Centre for Cancer, The Chinese University of Hong Kong, Hong Kong, China; Queen Mary Hospital, Hong Kong, China; Biostatistics, Millennium Pharmaceuticals, Inc., Cambridge, MA, USA, a wholly owned subsidiary of Takeda Pharmaceutical Company Limited, Cambridge, MA USA; Oncology Clinical Research, Millennium Pharmaceuticals, Inc., Cambridge, MA, USA, a wholly owned subsidiary of Takeda Pharmaceutical Company Limited, Cambridge, MA USA; Cancer Science Institute, National University of Singapore, Singapore, Republic of Singapore; National University Cancer Institute, National University Health System, Singapore, Republic of Singapore

**Keywords:** Multiple myeloma, Ixazomib, Ethnicity, East Asian, Pharmacokinetics

## Abstract

**Background:**

The oral proteasome inhibitor ixazomib is under phase 3 clinical investigation in multiple myeloma (MM) in combination with lenalidomide–dexamethasone. This study was conducted to investigate the pharmacokinetic and safety profiles of ixazomib, administered with lenalidomide–dexamethasone, in East Asian patients with relapsed/refractory MM.

**Methods:**

Adult patients with measurable disease who had received 1–3 prior lines of therapy received oral ixazomib on days 1, 8, and 15, lenalidomide (25 mg) on days 1–21, and dexamethasone (40 mg) on days 1, 8, 15, and 22, in 28-day cycles. Primary objectives were to characterize ixazomib plasma pharmacokinetics, determine the recommended phase 2/3 dose, and evaluate safety and tolerability.

**Results:**

Forty-three patients were enrolled. No dose-limiting toxicities were reported for the first six patients receiving ixazomib (4.0 mg), confirming this as the recommended phase 2/3 dose. Ixazomib was rapidly absorbed with a median *T*_max_ of 1.5 h on day 1 and 2.0 h on day 15 of cycle 1 and had a geometric mean terminal half-life of 6.1 days. Twenty-one (49 %) patients had at least one drug-related grade ≥3 adverse event (AE); the most common were neutropenia (19 %), diarrhea (14 %), and thrombocytopenia (12 %). Twenty-eight of 43 (65 %) response-evaluable patients had at least a partial response. The recommended phase 2/3 dose for ixazomib was determined to be 4.0 mg.

**Conclusions:**

The all-oral combination of ixazomib plus lenalidomide–dexamethasone appeared active and well tolerated at 4.0 mg. Consequently, East Asian patients enrolled in phase 3 studies are receiving the same ixazomib dose as patients in other regions.

**Trial registration:**

This study is registered at NCT01645930.

**Electronic supplementary material:**

The online version of this article (doi:10.1186/s13045-015-0198-1) contains supplementary material, which is available to authorized users.

## Background

Multiple myeloma (MM) is one of the most common hematologic malignancies, with approximately 86,000 new cases and 63,000 deaths reported globally each year [1], with recent reports suggesting that the incidence is increasing in some Asian countries [2]. The benefits of combining a proteasome inhibitor with an immunomodulatory drug and dexamethasone have been demonstrated in several clinical trials in patients with newly diagnosed MM (NDMM) [3–7] and in patients with relapsed and/or refractory MM (RRMM) [8–10], including Asian patients [11]. Substantial activity, including rapid and deep responses and prolonged progression-free survival, has been reported with bortezomib in combination with lenalidomide and dexamethasone and, more recently, carfilzomib plus lenalidomide and dexamethasone [5–7, 9, 10]. Following recent improvements in response rates and survival with the use of proteasome inhibitors and immunomodulatory drugs, and in the context of a growing interest in both maintenance and continuous therapy, there is an increased focus on long-term treatment and patient quality of life [12]. However, the feasibility of long-term treatment with current proteasome inhibitors may be limited for many patients due to toxicities or the need for regular clinic visits for intravenous/subcutaneous drug administration.

The investigational proteasome inhibitor ixazomib is the first orally administered proteasome inhibitor to be investigated in the clinic [13–16]. Phase 1 studies showed single-agent ixazomib to be generally well tolerated, with encouraging evidence of preliminary activity in patients with RRMM [13, 15]. The feasibility of combining ixazomib with lenalidomide and dexamethasone has been demonstrated in a phase 1/2 study in patients with NDMM; the results indicated a very high response rate (90 % overall response rate [ORR]) at the recommended phase 2/3 dose (RP2/3D) of ixazomib (4.0 mg), including 62 % very good partial response (VGPR) or better, with a manageable toxicity profile, including limited peripheral neuropathy (PN) [14]. On the basis of these early-phase data, phase 3 clinical trials are currently ongoing to assess the all-oral combination of weekly ixazomib (4.0 mg) plus lenalidomide and dexamethasone in patients with RRMM (NCT01564537) and NDMM (NCT01850524).

As the pharmacokinetic (PK) and safety profiles of a drug can be affected by ethnicity [17–19], this phase 1 PK study (NCT01645930) was conducted to investigate the PK and safety profiles of weekly oral ixazomib in combination with lenalidomide and dexamethasone and to determine the ixazomib RP2/3D specifically in East Asian patients with RRMM.

## Results

### Patients and treatment exposure

A total of 43 East Asian patients were enrolled. Patient baseline demographics and disease characteristics are shown in Table 1.

**Table 1:** Patient baseline demographics and disease characteristics

Characteristic	*N* = 43
Median age, years (range)	63 (38–79)
Male, *n* (%)	27 (63)
Asian ethnicity, *n* (%)	
Chinese	20 (47)
Korean	16 (37)
Other	7 (16)
ECOG performance status, *n* (%)	
0	24 (56)
1	17 (40)
2	2 (5)
ISS stage, *n* (%)	
I	10 (23)
II	18 (42)
III	13 (30)
Unknown	2 (5)
MM subtype, *n* (%)	
IgG	27 (63)
IgA	5 (12)
Kappa light chain	5 (12)
Lambda light chain	2 (5)
Biclonal	1 (2)
Unknown	3 (7)
Lytic bone lesions, *n* (%)	
Yes	32 (74)
No	10 (23)
Unknown	1 (2)
Median time since initial diagnosis to first dose of ixazomib, months (range)	36.3 (8–116)
Number of prior therapies, *n* (%)	
1	20 (47)
2	10 (23)
3	12 (29)
>3^a^	1 (2)
Prior therapy for MM, *n* (%)	
Corticosteroids	43 (100)
Bortezomib	32 (74)
Thalidomide	30 (70)
Lenalidomide	9 (21)
Carfilzomib	2 (5)
Prior transplant, *n* (%)	21 (49)

*ECOG* Eastern Cooperative Oncology Group, *ISS* International Staging System, *MM* multiple myeloma

^a^One patient had received five prior therapies

At the data cutoff (July 14, 2014), with enrollment completed and all patients having received at least 1 treatment cycle (i.e., completed the PK cycle of the study), the patients had received a median of 7 treatment cycles (range, 1–20 cycles), with 27 (63 %) and 6 (14 %) patients having received ≥6 and ≥12 cycles, respectively. A total of 22 patients (51 %) remained on treatment, having received a range of 1 to 20 treatment cycles. The mean relative dose intensity (proportion of dose prescribed that was actually taken) was 89.6 % for ixazomib, 68.7 % for lenalidomide, and 90.9 % for dexamethasone, with 12 (29 %), 5 (12 %), and 12 (28 %) of patients receiving 100 % of the planned dose of each drug, respectively; one patient received a lenalidomide starting dose of 15 mg, and four received a lenalidomide starting dose of 10 mg (all had creatinine clearance >50 mL/min).

### DLTs and determination of the recommended phase 2/3 dose

No dose-limiting toxicities (DLTs) were reported for the first six patients (three Chinese, three Indians) receiving the starting dose of 4.0 mg ixazomib. The RP2/3D of weekly ixazomib in combination with lenalidomide and dexamethasone was therefore determined as 4.0 mg, and all subsequently enrolled patients received 4.0 mg of ixazomib.

As the study continued, 2 of 24 DLT-evaluable patients experienced DLTs in cycle 1. One patient had drug-related grade 3 elevated alanine aminotransferase, grade 2 elevated alkaline phosphatase, and grade 2 elevated γ-glutamyltransferase, which, in the opinion of the investigator, were related to the study drugs (drug relationship was not reported for the three drugs separately); the DLTs were resolved following a treatment delay and the patient continued on study but received a reduced dose of lenalidomide (15 mg). A second patient, with a history of ongoing chronic atrophic gastritis and severe enterogastric reflux gastritis, had drug-related grade 3 diarrhea, which, in the opinion of the investigator, was related to lenalidomide; this DLT was resolved following patient withdrawal from the study.

### PK profile of ixazomib in combination with lenalidomide–dexamethasone

Ixazomib was rapidly absorbed with a median time to maximum plasma concentration (*T*_max_) of 1.5 h (range 0.5–8 h) on day 1 and 2.0 h (range 0.5–8 h) on day 15 of cycle 1 (Table 2). The overall geometric mean (% coefficient of variation [CV]) *C*_max_ (maximum observed plasma concentration) and AUC_0–168_ (area under the plasma concentration–time curve from 0 to 168 h post-dose) values on day 1 were 33.8 (115) ng/mL and 776 (78) hr*ng/mL, respectively. The corresponding values on day 15 were 43.9 (72) ng/mL and 1610 (48) hr*ng/mL, respectively.

**Table 2:** Plasma PK parameters of ixazomib on day 1 and day 15 of cycle 1

Parameter	Chinese	Korean	Other^a^	Overall
Day 1	*n* = 16	*n* = 16^b^	*n* = 6	*n* = 38^c^
*T*_max_, h^d^	1.04 (0.5–7.0)	1.95 (0.5–8.0)	1.75 (0.5–7.0)	1.5 (0.5–8.0)
*C*_max_, ng/mL	50.6 (102)	22.1 (99)	35.7 (118)	33.8 (115)
AUC_0–168_, h*ng/mL	933 (83)	630 (47)	720 (63)	776 (78)
DN *C*_max_, ng/mL/mg	12.6 (102)	5.53 (99)	8.93 (118)	8.45 (115)
DN AUC_0–168_, h*ng/mL/mg	233 (83)	157 (47)	180 (63)	194 (78)
Day 15	*n* = 15^e^	*n* = 13^f^	*n* = 4	*n* = 32^g^
*T*_max_, h^d^	2 (0.5–7.08)	2 (0.48–7.97)	1.25 (0.5–4.0)	2.0 (0.48–7.97)
*C*_max_, ng/mL	50.3 (71)	38.0 (67)	42.4 (97)	43.9 (72)
AUC_0–168_, h*ng/mL	1750 (39)	1450 (49)	1530 (81)	1610 (48)
DN *C*_max_, ng/mL/mg	12.6 (71)	9.51 (67)	10.6 (97)	11.0 (72)
DN AUC_0–168_, h*ng/mL/mg	438 (39)	362 (49)	383 (81)	401 (48)
t_1/2_, h	148 (20)	145 (20)	148 (7)	147 (18)
Accumulation ratio	2.38 (30)	2.34 (40)	2.56 (33)	2.39 (33)

Data are given as geometric mean (% CV), unless otherwise specified

*AUC*_*0–168*_ area under the plasma concentration–time curve from 0 to 168 h post-dose, *C*_max_ maximum observed plasma concentration, *DN* dose-normalized, *t*_*1/2*_ half-life of the terminal disposition phase, *T*_max_ time of C_max_

^a^Day 1: Malaysian (*n* = 3), Asian Indian (*n* = 2), Indonesian (*n* = 1); day 15: Malaysian (*n* = 3), Indonesian (*n* = 1)

^b^ *n* = 12 for AUC_0–168_

^c^ *n* = 34 for AUC_0–168_

^d^Median (range)

^e^ *n* = 14 for t_1/2_ and *n* = 12 for accumulation ratio

^f^ *n* = 11 for AUC_0–168_ and t_1/2_ and *n* = 9 for accumulation ratio

^g^ *n* = 30 for AUC_0–168,_ *n* = 29 for t_1/2_, and *n* = 25 for accumulation ratio

**Fig. 1:**
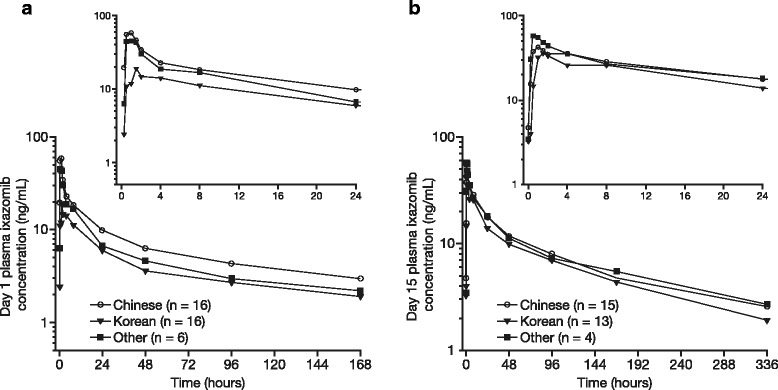
Mean plasma concentration–time profiles of ixazomib in combination with lenalidomide–dexamethasone on **a** day 1 and **b** day 15 of cycle 1 (safety population)

**Fig. 2:**
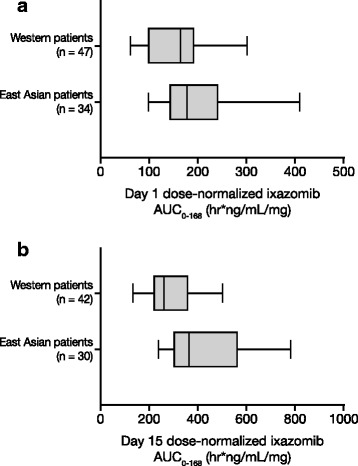
Ixazomib dose-normalized AUC_0–168_ on day 1 (**a**) and day 15 (**b**) of cycle 1 in East Asian and Western patients. *Box plots* represent the 25th, 50th, and 75th percentiles; *whiskers* denote the 10th and 90th percentiles. East Asian patients received ixazomib in combination with lenalidomide–dexamethasone. Western patients received ixazomib as a single agent or in combination with lenalidomide–dexamethasone

After multiple dosing, the terminal half-life was 6.1 days and the accumulation ratio (day 15 AUC_0–168_/day 1 AUC_0–168_) was 2.4 (Table 2). Mean plasma concentration–time profiles on days 1 and 15 are shown in Fig. 1; the PK profiles appeared similar across the different East Asian subgroups. The dose-normalized (DN) AUC data for East Asian patients from this study compared with Western patients (pooled data from three phase 1/2 trials of patients with RRMM (NCT00963820) [13], NDMM (NCT01217957) [14], and relapsed/refractory amyloid light-chain (AL) amyloidosis (NCT01318902) [16]) are shown in Fig. 2.

### Safety

All 43 patients received at least one dose of ixazomib plus lenalidomide–dexamethasone and were included in the safety population. All patients had at least one treatment-emergent adverse event (TEAE), and 31 (72 %) had at least one grade ≥3 TEAE (Table 3). Drug-related TEAEs and drug-related grade ≥3 TEAEs were seen in 37 (86 %) and 21 (49 %) patients, respectively.

**Table 3:** Safety profile of ixazomib plus lenalidomide–dexamethasone, overall and by East Asian ethnicity

AE, *n* (%)	Chinese	Korean	Other	Overall
	(*n* = 20)	(*n* = 16)	(*n* = 7)	(*N* = 43)
Any AE	20 (100)	16 (100)	7 (100)	43 (100)
Any drug-related AE	19 (95)	11 (69)	7 (100)	37 (86)
Any grade ≥3 AE	12 (60)	12 (75)	7 (100)	31 (72)
Any drug-related grade ≥3 AE	7 (35)	8 (50)	6 (86)	21 (49)
Any SAE^a^	8 (40)	5 (31)	5 (71)	18 (42)
Any drug-related SAE	3 (15)	4 (25)	3 (43)	10 (23)
AE resulting in any study drug dose reduction, *n* (%)	12 (60)	7 (44)	5 (71)	24 (56)
AE leading to discontinuation, *n* (%)	4 (20)	2 (13)	1 (14)	7 (16)
On-study deaths, *n* (%)	0	0	0	0

*AE* adverse event, *SAE* serious adverse event

^a^Defined as any AE or adverse reaction that results in death, is life-threatening, requires hospitalization or prolongation of existing hospitalization, results in persistent or significant disability or incapacity, or is a congenital anomaly or birth defect

The most common any grade and drug-related grade ≥3 TEAEs are shown in Table 4; the most common grade ≥3 drug-related AEs included neutropenia (*n* = 8, 19 %), thrombocytopenia (*n* = 5, 12 %), and diarrhea (*n* = 6, 14 %). Five (12 %) patients had a grade 4 drug-related TEAE; the most common of which was thrombocytopenia (four patients, 9 %), followed by hypokalemia, neutropenia, and renal impairment (one patient each, 2 %). The case of renal impairment improved following treatment; however, the patient subsequently developed pneumonia and died off-study, 42 days after receiving the last dose of study drug.

**Table 4:** Most common any grade (>10 % of patients) and grade ≥3 (>1 patient) drug-related AEs

	Chinese (*n* = 20)	Korean (*n* = 16)	Other (*n* = 7)	Overall (*N* = 43)
Any grade AE, *n* (%)				
Skin/subcutaneous tissue disorders^a^	10 (50)	4 (25)	7 (100)	21 (49)
Diarrhea	10 (50)	4 (25)	3 (43)	17 (40)
PN NEC^b^	4 (20)	3 (19)	4 (57)	11 (26)
Vomiting	4 (20)	3 (19)	2 (29)	9 (21)
Neutropenia	3 (15)	4 (25)	1 (14)	8 (19)
Thrombocytopenia	2 (10)	4 (25)	2 (29)	8 (19)
Decreased appetite	5 (25)	0	3 (43)	8 (19)
Fatigue	2 (10)	1 (6)	4 (57)	7 (16)
Nausea	2 (10)	4 (25)	1 (14)	7 (16)
Decreased platelet count	3 (15)	2 (13)	0	5 (12)
Insomnia	3 (15)	0	2 (29)	5 (12)
Grade ≥3 AE, *n* (%)				
Neutropenia	3 (15)	4 (25)	1 (14)	8 (19)
Thrombocytopenia	1 (10)	2 (25)	2 (29)	5 (12)
Diarrhea	0	4 (25)	2 (29)	6 (14)
Fatigue	1 (5)	1 (6)	2 (29)	4 (9)
Anemia	1 (5)	0	2 (29)	3 (7)
Hypokalemia	1 (5)	0	2 (29)	3 (7)

*AE* adverse event, *PN NEC* peripheral neuropathy not elsewhere classified

^a^System organ class, includes the preferred terms dry skin, macular rash, pruritus, skin hyperpigmentation (each *n* = 4), generalized pruritus, pruritic rash (each *n* = 3), maculopapular rash, skin exfoliation (each *n* = 2), skin discoloration, dermatitis acneiform, night sweats, drug eruption, papular rash, pigmentation disorder, and abnormal hair growth (each *n* = 1)

^b^Higher level term, includes the preferred terms peripheral neuropathy (*n* = 9) and peripheral sensory neuropathy (*n* = 2)

Drug-related TEAEs in the skin and subcutaneous tissue disorders, MedDRA System Organ Class, were reported in 21 (49 %) patients (Table 4), including drug hypersensitivity (grade 3 maculopapular rash) in one patient; this event was considered a serious drug-related TEAE and led to discontinuation of lenalidomide. All other rash-related events were grade ≤2 in intensity, and none was serious. Drug-related PN not elsewhere classified was reported in 11 (26 %) patients. This was grade 1 in eight patients (19 %) and grade 2 in three patients (7 %); there were no reports of grade ≥3 PN. Most (11/16, 67 %) cases of PN and peripheral sensory neuropathy were unresolved at the time of data cutoff. None of the neuropathy events was serious. There were two cardiac AEs: one patient had grade 1 angina pectoris, and one had grade 1 palpitations, neither was considered related to any of the study drugs and both were resolved within 3 months.

A total of 24 patients (56 %) had TEAEs requiring dose reduction of at least one of the study drugs. The most common were thrombocytopenia (seven patients, 16 %), fatigue, neutropenia, increased blood creatinine (each in three patients, 7 %), diarrhea, and PN (each in two patients, 5 %). Seven patients had TEAEs leading to discontinuation of at least one of the study drugs; these included pneumonia (two patients), acute renal impairment, plasmacytoma due to progressive disease, drug hypersensitivity, thrombocytopenia, spinal cord compression due to progressive disease, and worsening of PN (each in one patient). Ten (23 %) patients had at least one drug-related serious AE (SAE); diarrhea (three patients, 7 %) and pneumonia (two patients, 5 %) were the only drug-related SAEs seen in >1 patient. There were no on-study deaths.

### Response

To date, 28 of 43 response-evaluable patients have had a response (confirmed or unconfirmed), giving an ORR (PR or better) of 65 %. Ten (23 %) patients achieved ≥VGPR, including four (9 %) who attained a complete response (CR). A further five (12 %) patients had stable disease. Thirty (83 %) of the 36 patients in the safety population with measureable M-protein data had a >25 % decrease in the best percent change in M-protein, and 28 (78 %) patients had a >50 % decrease (Additional file 1: Figure S1). To date, of the 23 responders with a confirmed response, six have progressed, with a median duration of response of 12.9 months.

## Discussion

This is the first report of the all-oral combination of weekly ixazomib plus lenalidomide–dexamethasone in East Asian patients with RRMM. There were no DLTs in the first six patients who received the starting ixazomib dose of 4.0 mg, and the recommended weekly ixazomib dose was thus determined as 4.0 mg per protocol, the same as reported in Western patients [13, 14, 20]. The safety profile in East Asian patients also appears comparable to that reported in Western patients with NDMM who received ixazomib (4.0 mg) in combination with lenalidomide–dexamethasone [14]. Furthermore, preliminary efficacy data indicate that the regimen could be active in East Asian patients with RRMM. On the basis of the PK, safety, and preliminary efficacy data reported here, Asian patients are eligible for enrollment in the ongoing ixazomib phase 3 studies and will receive the same dose as administered in other regions.

When administered in combination with lenalidomide–dexamethasone in East Asian patients, ixazomib was rapidly absorbed with a median time to maximum plasma concentration of about 1.5–2 h. As seen in previous PK studies of ixazomib [13–15, 21], the long terminal half-life of ixazomib in this study (geometric mean of 6.1 days) supports weekly dosing. As reported for other agents [22, 23], the PK profile of ixazomib administered in combination with lenalidomide–dexamethasone was largely similar across the different East Asian races (e.g., Chinese, Korean), indicating no regional differences according to ethnicity and supporting the use of the same dose across East/North Asia. The PK profile reported here appears largely similar to that reported in a phase 1 study of ixazomib plus lenalidomide–dexamethasone in Western NDMM patients (NCT01217957) [14]. When pooling data from three studies in Western patients [13, 14, 24], ixazomib geometric mean DN AUC_0–168_ was found to be 28 % higher than East Asian RRMM patients on day 1 of dosing (194 [78 % CV] vs 152 [95 % CV] ng*h/mL/mg, respectively) and 49 % higher on day 15 (401 [48 % CV] vs 269 [44 % CV] ng*h/mL/mg, respectively). However, these increases in geometric mean exposure in East Asian patients are modest when viewed in the context of associated PK variability. Importantly, ixazomib exposures in Asian patients at the global phase 3 dose of 4.0 mg are not expected to exceed the exposures at the MTD in Western patients (2.97 mg/m^2^, which equates to 5.5 mg) [20], consistent with the safety and tolerability findings reported here with only 2 of 24 DLT-evaluable patients experiencing DLTs in cycle 1. Future population PK analyses will statistically evaluate the potential contribution of race to overall PK variability.

Overall, the regimen appears well tolerated in East Asian patients with RRMM. As seen in a phase 1 study of ixazomib plus lenalidomide–dexamethasone in Western patients [14], AEs were generally manageable with clinical intervention or dose modification. Consistent with the results in Western patients with NDMM [14], the most common drug-related grade ≥3 AEs were hematologic AEs, most commonly neutropenia and thrombocytopenia (seen in 19 % and 12 % of East Asian patients and 14 % and 8 % of Western patients, respectively), in both patient populations. Overall, median changes from baseline in neutrophil and platelet counts were generally small and not clinically relevant; during the first 3 cycles, a rapid decrease in platelets was observed at the beginning of the cycle followed by recovery before the next cycle. Also consistent with previous reports on the safety profile of ixazomib in Western patients [13–15, 21], but in contrast to data for the bortezomib–lenalidomide–dexamethasone triplet regimen (53 % sensory neuropathy, 17 % neuropathic pain, and 14 %, including 3 % grade 3, motor neuropathy) [9], the incidence of PN was low (26 %), and there were no reports of grade ≥3 PN. The tolerability of this regimen also compares favorably to that reported for the carfilzomib–lenalidomide–dexamethasone triplet regimen in patients with RRMM following 1–3 prior therapies [10], with similar low rates of PN but no drug-related cardiac toxicities.

The all-oral combination of ixazomib plus lenalidomide–dexamethasone has demonstrated encouraging anti-tumor activity in a phase 1/2 study in Western patients with NDMM. Following these early-phase data, the regimen is being investigated in two phase 3 studies in patients with MM: TOURMALINE-MM1 (NCT01564537), investigating weekly oral ixazomib or placebo plus lenalidomide–dexamethasone in patients with RRMM, and TOURMALINE-MM2 (NCT01850524), investigating weekly oral ixazomib or placebo plus lenalidomide–dexamethasone in patients with NDMM who are transplant-ineligible. This phase 1 study confirmed that 4.0 mg ixazomib, the phase 3 dose for Western patients, combined with lenalidomide and dexamethasone, is effective and well tolerated in Asian patients with RRMM. The RP2/3D for ixazomib combined with lenalidomide and dexamethasone is 4.0 mg in Asian patients with RRMM.

## Methods

### Patients

East Asian patients aged ≥18 years with RRMM following 1–3 prior lines of therapy, measurable disease (serum M-protein ≥1 g/dL, urine M-protein ≥200 mg/24 h, or involved free light chain ≥10 mg/dL), Eastern Cooperative Oncology Group performance status 0–2, and adequate hepatic (bilirubin ≤1.5 × upper limit of normal [ULN], alanine aminotransferase and aspartate aminotransferase ≤3 × ULN), renal (calculated creatinine clearance ≥30 mL/min), and hematologic (absolute neutrophil count ≥1000/mm^3^, platelet count ≥75,000/mm^3^) function were eligible. Patients with grade ≥2 PN or grade 1 PN with pain, or any co-morbid systemic illness or other severe concurrent disease that, in the judgment of the investigator, would make the patient inappropriate for entry into the study or interfere significantly with the proper assessment of safety and toxicity of the prescribed regimens were not eligible. Patients who received systemic treatment, within 14 days before study enrollment, with strong inhibitors of CYP1A2, strong inhibitors of CYP3A, or strong CYP3A inducers, or used *Ginkgo biloba* or St. John’s wort were also not eligible.

### Study design

The primary objectives of this multicenter, phase 1 PK, tolerability, and dose-determination study were to characterize the plasma PK parameters of ixazomib administered in combination with lenalidomide and low-dose dexamethasone, determine the RP2/3D, and evaluate the safety and tolerability in East Asian patients. Secondary objectives included evaluation of the ORR, defined as partial response or better.

The patients received oral ixazomib weekly on days 1, 8, and 15, lenalidomide (25 mg) on days 1–21, and dexamethasone (40 mg) on days 1, 8, 15, and 22, in 28-day cycles until progressive disease or unacceptable toxicity. Ixazomib was taken at least 1 h before or 2 h after a meal; when applicable, ixazomib and lenalidomide were taken at the same time. Dexamethasone was taken at least 1 h after ixazomib and lenalidomide administration. Lenalidomide starting dose was adjusted for patients with renal impairment based on local prescribing information. All patients received mandatory thromboprophylaxis with aspirin, low-molecular-weight heparin, or an alternative agent according to the American Society of Clinical Oncology guidelines [25] or institutional standards of care, and as required by the lenalidomide prescribing information label. Dose modifications could be made based on toxicities. Concomitant administration of strong inhibitors of CYP1A2, strong inhibitors of CYP3A, or *G. biloba* or St. John’s wort was not permitted; concomitant administration of strong CYP3A inducers was to be avoided.

DLTs were defined as one or more of the following toxicities considered related to therapy: (1) grade 4 neutropenia lasting ≥7 days or grade 3 neutropenia with fever (≥38.5 °C) and/or infection; (2) grade 4 thrombocytopenia lasting ≥7 days, grade 3 thrombocytopenia with clinically significant bleeding, or platelets <10 000/mm^3^ at any time; (3) any grade ≥3 non-hematologic toxicity except grade 3 arthralgia/myalgia and brief (<1 week) grade 3 fatigue; (4) grade 2 PN with pain; (5) a delay of ≥2 weeks in starting cycle 2 due to lack of recovery from ixazomib-related toxicities in cycle 1; or (6) other study drug-related grade ≥2 non-hematologic toxicities requiring drug discontinuation. DLTs were assessed in cycle 1, and DLTs observed during cycle 1 in the first six patients were used to determine the tolerable ixazomib dose for the study. The starting dose of ixazomib was 4.0 mg (the RP2/3D in Western patients with NDMM [14, 20]), with planned dose de-escalation to 3.0 mg and then 2.3 mg, following a standard 3 + 3 design, if the starting dose was not acceptably tolerated in East Asian patients (Additional file 1: Figure S2).

All patients provided written informed consent, and review boards at all participating centers approved the study protocol and protocol amendments, and the trial was conducted according to the stipulations set out in the Declaration of Helsinki and International Conference on Harmonisation Guideline for Good Clinical Practice. The study was registered at www.clinicaltrials.gov as NCT01645930.

### Assessments

Blood samples for PK analysis of ixazomib were collected at prespecified time points during cycle 1 (within 1 h pre-dose and 0.25, 0.5, 1, 1.5, 2, 4, 8, 24, 48, 96, and 168 h post-dose on days 1 and 15) and within 1 h prior to dosing on day 1 of cycle 2. Ixazomib plasma concentrations were measured using a validated liquid chromatography/tandem mass spectrometry (LC/MS/MS) assay. AEs were evaluated throughout and up to 30 days after the last dose of study medication and were graded according to the National Cancer Institute Common Terminology Criteria for Adverse Events (NCI-CTCAE) v4.03. Response was assessed by investigators every other cycle using the International Myeloma Working Group uniform response criteria [26].

### Statistical analyses

All patients who received at least one dose of study drug were included in the safety population; those who also had at least one post-treatment response evaluation were included in the response-evaluable population. The DLT-evaluable population included all patients who had a DLT during cycle 1 or received all scheduled doses and completed all study procedures in cycle 1 without having a DLT.

Plasma PK parameters were calculated for patients in the safety population following administration of ixazomib on cycle 1 day 1 and/or cycle 1 day 15 provided sufficient concentration–time data were available, the patient had received the protocol-specified ixazomib dosing regimen during cycle 1, and no excluded concomitant medications were received during the PK sampling period. PK parameters were estimated using non-compartmental analysis methods from the ixazomib concentration versus time data. The analyses were performed using Phoenix WinNonlin version 6.2 (Pharsight, Princeton, NJ). PK parameters were summarized, as appropriate, using descriptive statistics. DN AUC data in East Asian patients were compared with data for Western patients, pooled from three phase 1/2 trials of patients with RRMM (NCT00963820) [13], NDMM (NCT01217957) [14], and relapsed/refractory AL amyloidosis (NCT01318902) [16].

## Additional file

Additional file 1: Figure S1. Waterfall plot of the best percent change in M-protein from baseline. **Figure S2.** Flowchart for ixazomib dose determination during cycle 1.

## References

[1] Becker N (2011). Epidemiology of multiple myeloma. Recent Results Cancer Res.

[2] Kim K, Lee JH, Kim JS, Min CK, Yoon SS, Shimizu K (2014). Clinical profiles of multiple myeloma in Asia-An Asian Myeloma Network study. Am J Hematol.

[3] Cavo M, Tacchetti P, Patriarca F, Petrucci MT, Pantani L, Galli M (2010). Bortezomib with thalidomide plus dexamethasone compared with thalidomide plus dexamethasone as induction therapy before, and consolidation therapy after, double autologous stem-cell transplantation in newly diagnosed multiple myeloma: a randomised phase 3 study. Lancet.

[4] Cavo M, Pantani L, Petrucci MT, Patriarca F, Zamagni E, Donnarumma D (2012). Bortezomib-thalidomide-dexamethasone is superior to thalidomide-dexamethasone as consolidation therapy after autologous hematopoietic stem cell transplantation in patients with newly diagnosed multiple myeloma. Blood.

[5] Jakubowiak AJ, Dytfeld D, Griffith KA, Lebovic D, Vesole DH, Jagannath S (2012). A phase 1/2 study of carfilzomib in combination with lenalidomide and low-dose dexamethasone as a frontline treatment for multiple myeloma. Blood.

[6] Kumar S, Flinn I, Richardson PG, Hari P, Callander N, Noga SJ (2012). Randomized, multicenter, phase 2 study (EVOLUTION) of combinations of bortezomib, dexamethasone, cyclophosphamide, and lenalidomide in previously untreated multiple myeloma. Blood.

[7] Richardson PG, Weller E, Lonial S, Jakubowiak AJ, Jagannath S, Raje NS (2010). Lenalidomide, bortezomib, and dexamethasone combination therapy in patients with newly diagnosed multiple myeloma. Blood.

[8] Garderet L, Iacobelli S, Moreau P, Dib M, Lafon I, Niederwieser D (2012). Superiority of the triple combination of bortezomib-thalidomide-dexamethasone over the dual combination of thalidomide-dexamethasone in patients with multiple myeloma progressing or relapsing after autologous transplantation: the MMVAR/IFM 2005–04 Randomized Phase III Trial from the Chronic Leukemia Working Party of the European Group for Blood and Marrow Transplantation. J Clin Oncol.

[9] Richardson PG, Xie W, Jagannath S, Jakubowiak A, Lonial S, Raje NS (2014). A phase 2 trial of lenalidomide, bortezomib, and dexamethasone in patients with relapsed and relapsed/refractory myeloma. Blood.

[10] Stewart AK, Rajkumar SV, Dimopoulos MA, Masszi T, Spicka I, Oriol A (2015). Carfilzomib, lenalidomide, and dexamethasone for relapsed multiple myeloma. N Engl J Med.

[11] Eom HS, Kim YK, Chung JS, Kim K, Kim HJ, Kim HY (2010). Bortezomib, thalidomide, dexamethasone induction therapy followed by melphalan, prednisolone, thalidomide consolidation therapy as a first line of treatment for patients with multiple myeloma who are non-transplant candidates: results of the Korean Multiple Myeloma Working Party (KMMWP). Ann Hematol.

[12] Rollig C, Knop S, Bornhauser M (2014). Multiple myeloma. The Lancet.

[13] Kumar SK, Bensinger WI, Zimmerman TM, Reeder CB, Berenson JR, Berg D (2014). Phase 1 study of weekly dosing with the investigational oral proteasome inhibitor ixazomib in relapsed/refractory multiple myeloma. Blood.

[14] Kumar SK, Berdeja JG, Niesvizky R, Lonial S, Laubach JP, Hamadani M (2014). Safety and tolerability of ixazomib, an oral proteasome inhibitor, in combination with lenalidomide and dexamethasone in patients with previously untreated multiple myeloma: an open-label phase 1/2 study. Lancet Oncol.

[15] Richardson PG, Baz R, Wang M, Jakubowiak AJ, Laubach JP, Harvey RD (2014). Phase 1 study of twice-weekly ixazomib, an oral proteasome inhibitor, in relapsed/refractory multiple myeloma patients. Blood.

[16] Merlini G, Sanchorawala V, Jeffrey ZA, Kukreti V, Schoenland SO, Jaccard A (2014). Long-term outcome of a phase 1 study of the investigational oral proteasome inhibitor (PI) ixazomib at the recommended phase 3 dose (RP3D) in patients (Pts) with relapsed or refractory systemic light-chain (AL) amyloidosis (RRAL). Blood.

[17] Johnson JA (2000). Predictability of the effects of race or ethnicity on pharmacokinetics of drugs. Int J Clin Pharmacol Ther.

[18] Yasuda SU, Zhang L, Huang SM (2008). The role of ethnicity in variability in response to drugs: focus on clinical pharmacology studies. Clin Pharmacol Ther.

[19] Ramamoorthy A, Pacanowski M, Bull J, Zhang L (2015). Racial/ethnic differences in drug disposition and response: review of recently approved drugs. Clin Pharmacol Ther.

[20] Gupta N, Zhao Y, Hui AM, Esseltine DL, Venkatakrishnan K (2014). Switching from body surface area-based to fixed dosing for the investigational proteasome inhibitor ixazomib: a population pharmacokinetic analysis. Br J Clin Pharmacol.

[21] Assouline SE, Chang J, Cheson BD, Rifkin R, Hamburg S, Reyes R (2014). Phase 1 dose-escalation study of IV ixazomib, an investigational proteasome inhibitor, in patients with relapsed/refractory lymphoma. Blood Cancer J.

[22] Myrand SP, Sekiguchi K, Man MZ, Lin X, Tzeng RY, Teng CH (2008). Pharmacokinetics/genotype associations for major cytochrome P450 enzymes in native and first- and third-generation Japanese populations: comparison with Korean, Chinese, and Caucasian populations. Clin Pharmacol Ther.

[23] Oishi M, Hiro S, Matsuoka N, Hotta S, Ono R, Mori Y (2014). A comparison of the pharmacokinetics and drug safety among East Asian populations. Ther Innov Regul Sci.

[24] Merlini G, Sanchorawala V, Zonder J A, Kukreti V, Schoenland S O, Jaccard A, et al. MLN9708, a novel, investigational oral proteasome inhibitor, in patients with relapsed or refractory light-chain amyloidosis (AL): Results of a Phase 1 Study. http://myeloma.org/pdfs/Merlini-731-3876.pdf. Accessed January 20, 2015.

[25] Lyman GH, Khorana AA, Kuderer NM, Lee AY, Arcelus JI, Balaban EP (2013). Venous thromboembolism prophylaxis and treatment in patients with cancer: American Society of Clinical Oncology clinical practice guideline update. J Clin Oncol.

[26] Durie BG, Harousseau JL, Miguel JS, Blade J, Barlogie B, Anderson K (2006). International uniform response criteria for multiple myeloma. Leukemia.

