# Supplementary material for: Pharmacokinetics and safety of ixazomib plus lenalidomide–dexamethasone in Asian patients with relapsed/refractory myeloma: a phase 1 study
Source: J Hematol Oncol. 2015 Sep 4;8:103. doi: 10.1186/s13045-015-0198-1 (PMC4559079; doi:10.1186/s13045-015-0198-1)
Supplement: Additional file 1: Figure S1. — Waterfall plot of the best percent change in M-protein from baseline. Figure S2. Flowchart for ixazomib dose determination during cycle 1. [file 13045_2015_198_MOESM1_ESM.docx]

**Additional file 1**

**Figure S1.** Waterfall plot of best percent change in M-protein from baseline


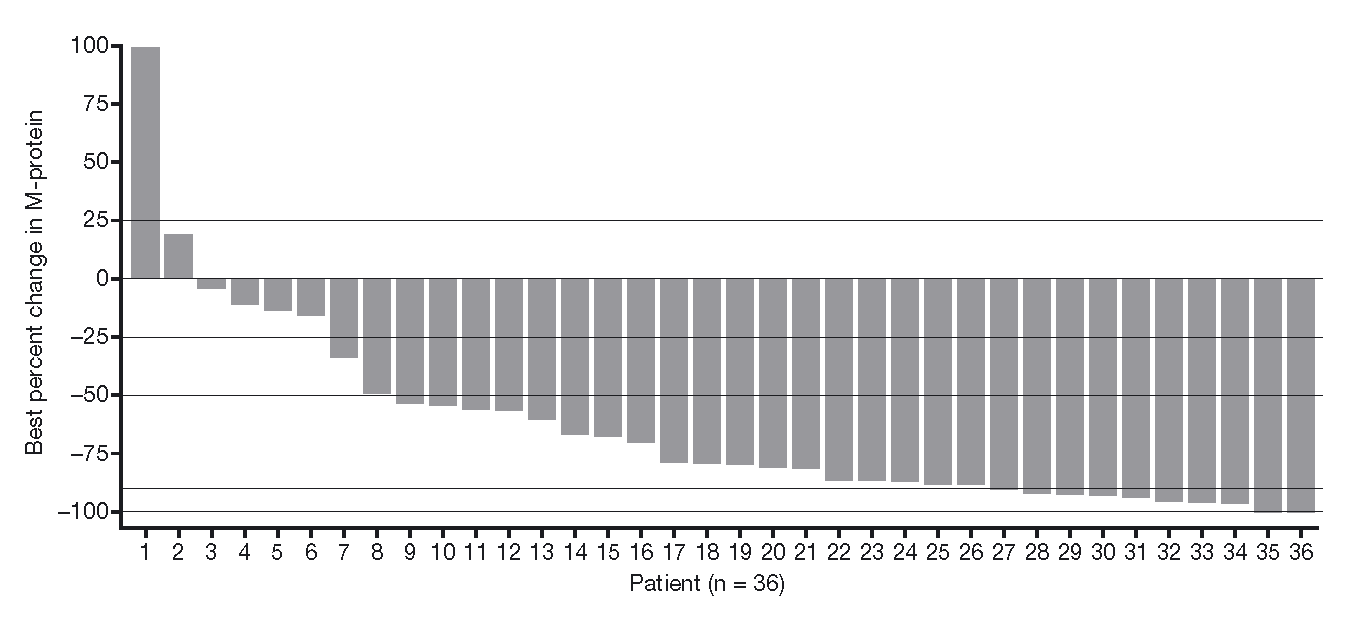


**Figure S2.** Flowchart for ixazomib dose determination during cycle 1.

**
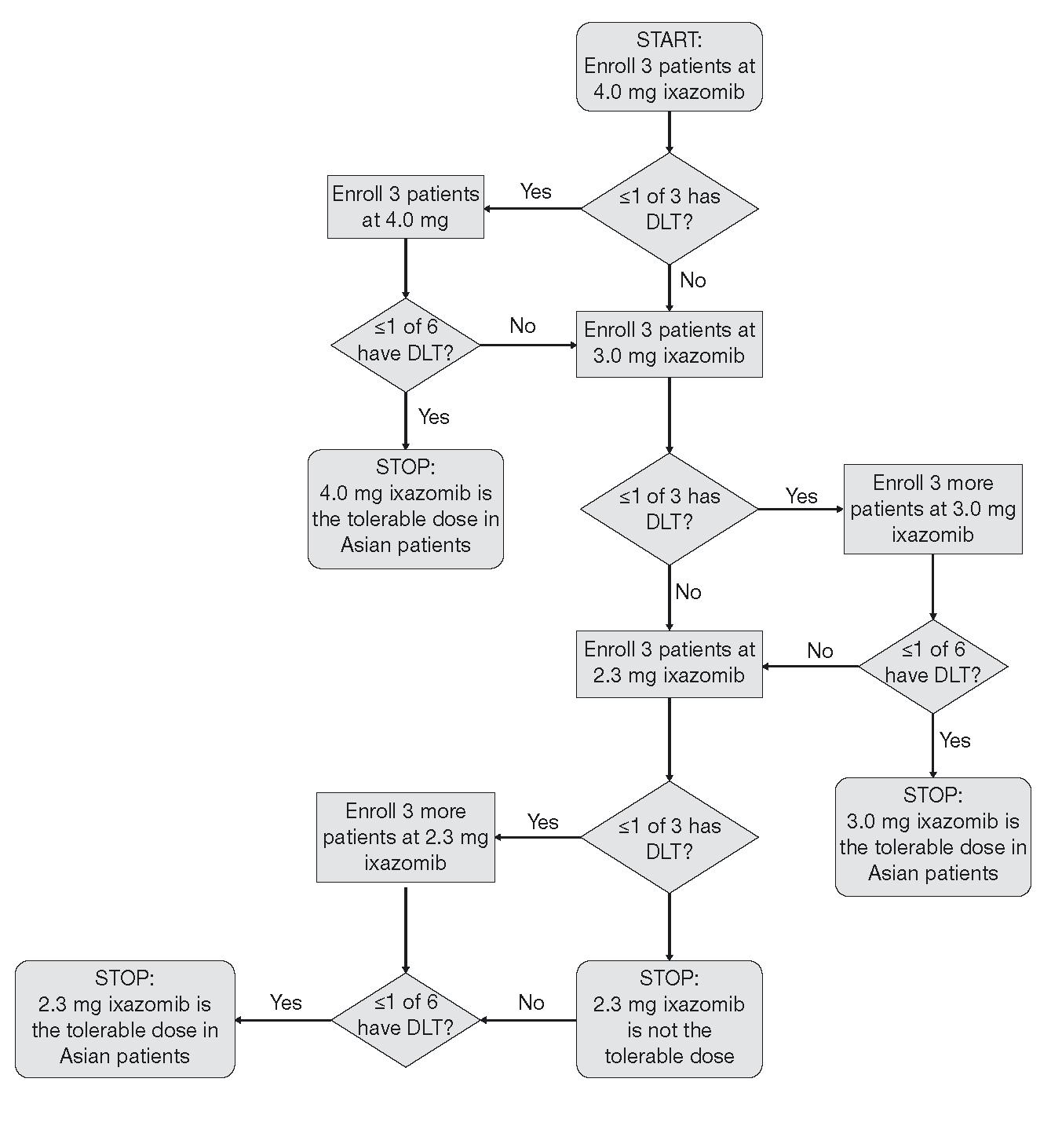
**
